# Selective ARID1A Loss Restricted to the Undifferentiated Component of a Mismatch Repair‐Deficient Gastric Carcinoma: A Case Report

**DOI:** 10.1155/crgm/8863202

**Published:** 2026-05-05

**Authors:** Rika Omote, Ryosuke Hamano, Shinya Otsuka, Takehiro Tanaka, Hiroyuki Yanai

**Affiliations:** ^1^ Department of Diagnostic Pathology, NHO Fukuyama Medical Center, Hiroshima, Japan; ^2^ Department of Surgery, NHO Fukuyama Medical Center, Hiroshima, Japan; ^3^ Department of Pathology, Okayama University Hospital, Okayama, Japan, okayama-u.ac.jp

**Keywords:** ARID1A, carcinoma, DNA mismatch repair deficiency, rhabdoid features, stomach neoplasms, SWI/SNF complex, undifferentiated

## Abstract

Mismatch repair‐deficient (dMMR) gastric carcinomas often harbor ARID1A alteration, but a sharply demarcated undifferentiated/rhabdoid component with selective ARID1A loss is uncommon and may create a diagnostic dilemma. An 80‐year‐old man underwent esophagogastroduodenoscopy for anemia, which revealed a circumferential Borrmann Type 3 lesion in the gastric body, and distal gastrectomy was performed. Histologically, the tumor was composed predominantly of undifferentiated carcinoma with focal rhabdoid features and a minute well‐differentiated adenocarcinoma component, with an abrupt transition between the two. Immunohistochemistry showed loss of nuclear MLH1 and PMS2 in both components, whereas loss of ARID1A expression was confined to the undifferentiated component; SMARCB1 (INI1), SMARCA2 (BRM), and SMARCA4 (BRG1) were retained. EBER in situ hybridization was negative. Because gene‐level testing, MSI testing, and MLH1 promoter methylation analysis were not performed, the molecular basis of the dMMR phenotype and ARID1A loss could not be determined. The restricted scope of molecular testing limits the ability to draw broad or generalizable conclusions and to fully establish clinicopathological correlations. The value of this report is, therefore, not mechanistic proof but recognition of a practical morphologic‐immunophenotypic observation: When a gastric carcinoma shows a sharply demarcated shift from differentiated to undifferentiated/rhabdoid morphology, dMMR should be considered, and selective ARID1A loss in the undifferentiated component may be associated with dedifferentiation. These findings should be interpreted with caution as preliminary, hypothesis‐generating observations that require validation in larger studies with more extensive molecular profiling.

## 1. Introduction

Gastric cancer remains a major global health burden, with 968,784 new cases worldwide in 2022 [1]. The Cancer Genome Atlas (TCGA) classifies gastric adenocarcinoma into four molecular subtypes: Epstein–Barr virus (EBV)–associated, microsatellite instability (MSI), genomically stable, and chromosomal instability [2, 3]. The dMMR/MSI subtype accounts for a minor but clinically distinctive subset of gastric cancers and is frequently associated with MLH1/PMS2 loss, often in relation to sporadic MLH1 promoter hypermethylation [4]. ARID1A, a component of the SWI/SNF chromatin‐remodeling complex, is altered preferentially in EBV‐associated and MSI/dMMR gastric carcinomas [5–7].

However, the morphologic implications of selective ARID1A loss within mixed differentiated/undifferentiated gastric carcinomas remain incompletely characterized. We report such a case to highlight a practical clinicopathologic pattern that may aid histologic interpretation and immunohistochemical workup. We also explicitly acknowledge that, in the absence of molecular testing, this case cannot establish the genomic basis of either the dMMR phenotype or ARID1A loss.

## 2. Case Presentation

**FIGURE 1:**
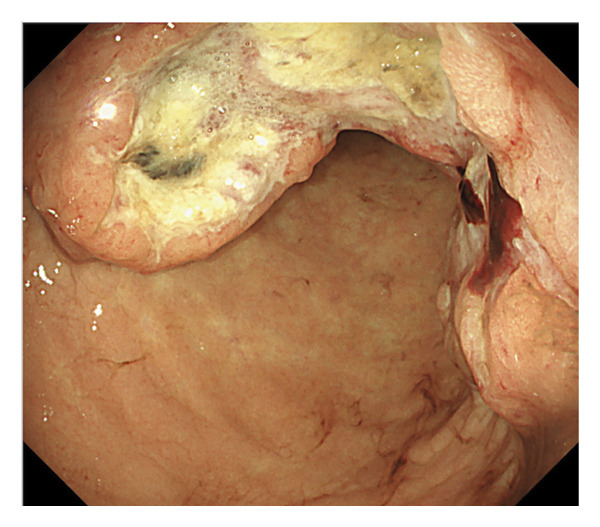
Esophagogastroduodenoscopy revealing a circumferential Borrmann Type 3 lesion in the gastric body.

An 80‐year‐old man was found to have anemia on routine laboratory testing by his primary care physician. Esophagogastroduodenoscopy revealed a circumferential Borrmann Type 3 lesion in the gastric body (Figure 1). Biopsy confirmed adenocarcinoma, and he was referred to our institution. Contrast‐enhanced computed tomography demonstrated enlarged regional lymph nodes without distant metastases. Distal gastrectomy with lymph node dissection was performed.

**FIGURE 2:**
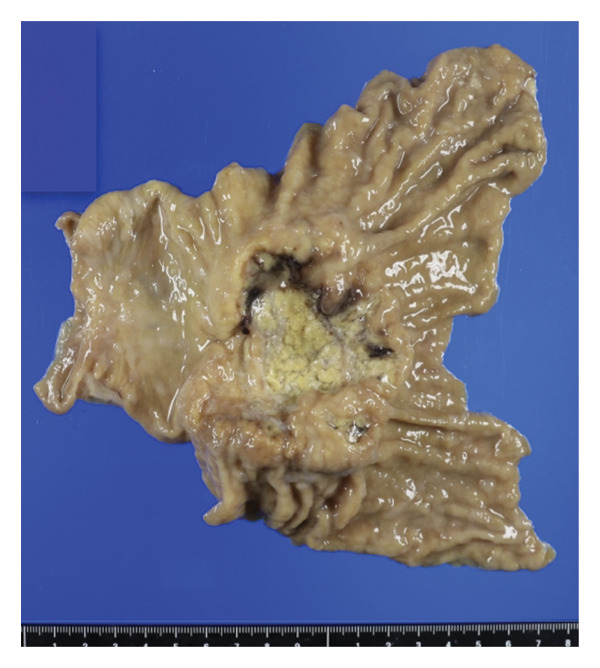
Gross examination showing a well‐demarcated ulcerated tumor measuring 5 cm.

**FIGURE 3:**
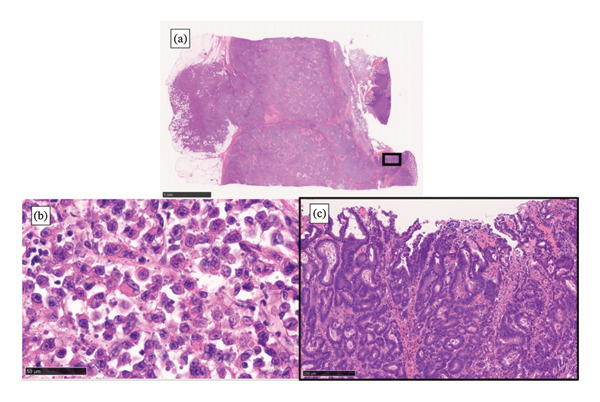
The lesion was predominantly undifferentiated carcinoma with focal rhabdoid features and a minute focus of well‐differentiated adenocarcinoma, showing an abrupt transition between the two components.

Gross examination showed a well‐demarcated ulcerated tumor measuring 5 cm (Figure 2). Microscopically, the lesion was composed predominantly of undifferentiated carcinoma with focal rhabdoid features and a minute focus of well‐differentiated adenocarcinoma, with an abrupt transition between the two components (Figure 3).

**FIGURE 4:**
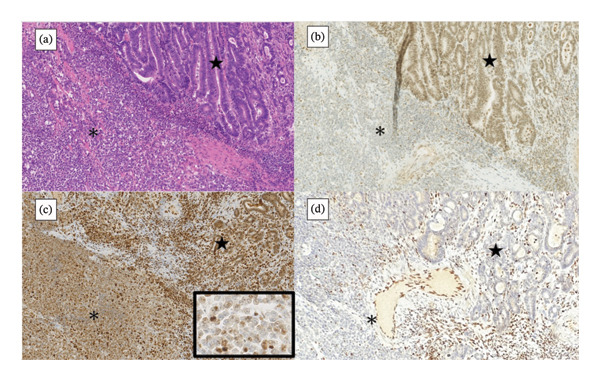
Representative immunohistochemistry demonstrating loss of MLH1 and PMS2 in both components and selective loss of ARID1A in the undifferentiated component. In the undifferentiated component (asterisk), ARID1A expression was lost. In the well‐differentiated adenocarcinoma component (star), ARID1A expression was retained, whereas MLH1 and PMS2 were absent in both components.

Immunohistochemically, both tumor components were positive for cytokeratin AE1/AE3. Neuroendocrine markers (CD56, synaptophysin, and chromogranin A) were negative, and CD20/CD3 findings did not support lymphoma. Tumor cells in both components showed loss of nuclear MLH1 and PMS2, with preserved staining in internal nonneoplastic cells. In contrast, ARID1A expression was lost only in the undifferentiated component and retained in the well‐differentiated adenocarcinoma component (Figure 4). SMARCB1 (INI1), SMARCA2 (BRM), and SMARCA4 (BRG1) were retained in both components. EBER‐ISH was negative. The immunophenotypic findings are summarized in Table 1.

**TABLE 1:** Immunohistochemical profile of the two tumor components.

Marker	Well‐differentiated component	Undifferentiated component
AE1/AE3	Positive	Positive
CD56	Negative	Negative
Synaptophysin	Negative	Negative
Chromogranin A	Negative	Negative
CD20	Negative	Negative
CD3	Negative	Negative
MLH1	Loss (dot‐like positive)	Loss (dot‐like positive)
PMS2	Loss	Loss
ARID1A	Retained	Loss
SMARCB1 (INI1)	Retained	Retained
SMARCA2 (BRM)	Retained	Retained
SMARCA4 (BRG1)	Retained	Retained

*Note:* “Loss” and “retained” refer to nuclear staining in tumor cells. Internal nonneoplastic cells served as positive controls for MLH1 and PMS2.

The postoperative pathologic stage was pT4aN2, stage IIIA. The patient declined both comprehensive genomic profiling and adjuvant chemotherapy and was transferred to a long‐term care hospital. According to follow‐up information obtained from the transfer hospital, the patient is alive without recurrence 30 months after surgery.

## 3. Discussion

This case is notable for an abrupt transition between a minute differentiated adenocarcinoma component and a predominant undifferentiated/rhabdoid component within a gastric carcinoma showing a dMMR immunophenotype in both areas, while ARID1A loss was restricted to the undifferentiated component. We believe that the principal value of the case lies in documenting a practical morphologic‐immunophenotypic observation rather than in establishing definitive clinicopathological or mechanistic conclusions.

The SWI/SNF chromatin‐remodeling complex, which includes ARID1A, SMARCB1, SMARCA2, and SMARCA4, helps regulate chromatin accessibility, lineage‐specific enhancer activity, and epithelial differentiation [8–12]. Loss of core subunits such as SMARCB1 or SMARCA4 can be associated with profoundly undifferentiated/rhabdoid morphology in gastrointestinal carcinomas [10–12]. By contrast, isolated ARID1A loss may be at least partially buffered by ARID1B, such that differentiated architecture can be preserved [13]. Concurrent ARID1A and ARID1B inactivation has been implicated in rapid dedifferentiation in gynecologic dedifferentiated carcinomas [14]. In the present case, retention of SMARCB1, SMARCA2, and SMARCA4 argues against canonical loss of those core subunits, whereas the selective ARID1A‐loss pattern supports an association between ARID1A loss and dedifferentiation. However, because ARID1B immunohistochemistry and genomic testing were not available, ARID1A loss alone cannot be regarded as a proven cause of the undifferentiated morphology in this tumor.

Undifferentiated carcinomas may coexist with glandular components, supporting dedifferentiation from a differentiated precursor [15]. Abrupt histologic transition within a gastric carcinoma has also been reported as a clue to dMMR/MSI‐high biology [16]. In our case, both components showed MLH1/PMS2 loss, while ARID1A loss was confined to the undifferentiated component. This distribution is compatible with, but does not prove, a stepwise process in which a dMMR background preceded secondary ARID1A loss during dedifferentiation. Because MSI testing was not performed, we interpret the case as immunohistochemically dMMR rather than molecularly confirmed MSI high.

Large cohort and meta‐analytic studies have shown that abnormal or lost ARID1A expression in gastric carcinoma is associated with adverse clinicopathologic features overall and is enriched in dMMR/MSI and EBV‐associated tumors [6, 7, 17]. At the same time, the prognostic effect of ARID1A appears to be pathway dependent, and its significance in MLH1‐lost tumors may differ from that in MLH1‐preserved/EBV‐negative tumors [7]. For this reason, and because follow‐up after transfer was limited to survival status and recurrence information at 30 months, we have avoided drawing any case‐specific prognostic implication from the present report.

Several limitations should be emphasized. First, no sequencing, MSI testing, germline evaluation, or MLH1 promoter methylation analysis was performed; accordingly, neither the genomic basis of the dMMR phenotype nor the mechanism of ARID1A loss can be resolved in this case. The ARID1A finding should therefore be interpreted as a protein‐expression abnormality by immunohistochemistry rather than a genetically proven ARID1A alteration. More broadly, the restricted scope of molecular testing substantially limits the ability to draw broad or generalizable conclusions and to fully establish clinicopathological correlations. Second, ARID1B immunohistochemistry was not performed, precluding more detailed interrogation of cBAF dysfunction. Third, follow‐up after transfer to a long‐term care facility was limited to survival status and recurrence information at 30 months, precluding more detailed longitudinal assessment. Thus, this report should be interpreted as documentation of a distinctive morphologic‐immunophenotypic pattern with practical diagnostic relevance. However, as a single case report, these observations remain preliminary and hypothesis‐generating and require validation in larger studies with more extensive molecular profiling.

## 4. Conclusion

When a gastric carcinoma shows an abruptly demarcated undifferentiated/rhabdoid component adjacent to differentiated adenocarcinoma, dMMR should be considered in the differential diagnosis. ARID1A immunostaining may be informative, particularly when loss is confined to the undifferentiated component. Such a pattern may be compatible with stepwise dedifferentiation within a dMMR gastric carcinoma. However, given the restricted molecular testing in this single case, the findings should be regarded as preliminary and hypothesis‐generating rather than broadly generalizable, and they require validation in larger studies with more comprehensive molecular profiling.

## Author Contributions

R.H. and S.O. collected clinical and pathologic data. R.O., T.T., and H.Y. drafted the manuscript. All authors critically revised the manuscript.

## Funding

This work received no specific grant from any funding agency in the public, commercial, or not‐for‐profit sectors.

## Disclosure

All authors read and approved the final version of the manuscript. This work was performed as part of the authors’ employment at National Hospital Organization Fukuyama Medical Center, Fukuyama, Japan. The employer was not involved in manuscript writing, editing, approval, or the decision to publish. This case report was prepared in accordance with the CARE guidelines.

## Ethics Statement

Ethical approval was not required for this single‐patient case report according to our institutional policy.

## Consent

Written informed consent for publication of the case details and accompanying images was obtained from the patient.

## Conflicts of Interest

The authors declare no conflicts of interest.

## Data Availability

All data supporting the findings of this case report are included in the article. Additional anonymized images are available from the corresponding author upon reasonable request.
